# The JAK-STAT Pathway as a Therapeutic Strategy in Cancer Patients with Immune Checkpoint Inhibitor-Induced Colitis: A Narrative Review

**DOI:** 10.3390/cancers16030611

**Published:** 2024-01-31

**Authors:** Antonietta Gerarda Gravina, Raffaele Pellegrino, Alfonso Esposito, Marina Cipullo, Mario Romeo, Giovanna Palladino, Patrizia Iodice, Alessandro Federico, Teresa Troiani

**Affiliations:** 1 Hepatogastroenterology Division, Department of Precision Medicine, University of Campania Luigi Vanvitelli, Via L. de Crecchio, 80138 Naples, Italy; 2 Oncology Division, Department of Precision Medicine, University of Campania Luigi Vanvitelli, Via L. de Crecchio, 80138 Naples, Italy; 3 Oncology Division, AORN Ospedali Dei Colli, Monaldi Hospital, Via L. Bianchi, 80131 Naples, Italy

**Keywords:** immunotherapy, immune checkpoint inhibitors, colitis, JAK-STAT, tofacitinib, immune-related adverse events

## Abstract

**Simple Summary:**

Clinical management of some cancers requires immunotherapy using immune checkpoint inhibitors (ICI). ICI, however, can result in severe gastrointestinal toxicity, including ICI colitis. The main guidelines recommend the use of corticosteroids or some biologic drugs such as infliximab or vedolizumab. However, little space is given to Janus kinase (JAK)–signal transducer and activator of transcription (STAT) inhibitors. This review summarizes the primary evidence for using JAK-STAT inhibitors in managing ICI colitis. Tofacitinib has been provided in multiple case reports of being able to induce solid clinical remission of ICI colitis without concerns for the oncologic progression of the underlying neoplasm under immunotherapy treatment.

**Abstract:**

Immunotherapy has emerged as a pivotal component in the treatment of various malignancies, encompassing lung, skin, gastrointestinal, and head and neck cancers. The foundation of this therapeutic approach lies in immune checkpoint inhibitors (ICI). While ICIs have demonstrated remarkable efficacy in impeding the neoplastic progression of these tumours, their use may give rise to substantial toxicity, notably in the gastrointestinal domain, where ICI colitis constitutes a significant aspect. The optimal positioning of Janus kinase (JAK)–signal transducer and activator of transcription (STAT) pathway inhibitors in the therapeutic management of ICI colitis remains unclear. Numerous reports have highlighted notable improvements in ICI colitis through the application of pan-JAK-STAT inhibitors, with tofacitinib, in particular, reporting evident clinical remission of colitis. The precise mechanism by which JAK-STAT inhibitors may impact the pathogenetic process of ICI colitis remains inadequately understood. However, there is speculation regarding their potential role in modulating memory resident CD8^+^ T lymphocytes. The elucidation of this mechanism requires further extensive and robust evidence, and ongoing JAK-STAT-based trials are anticipated to contribute valuable insights.

## 1. Introduction

Immunotherapy is indicated in several malignancies, such as lung cancer, colorectal, melanoma, and head and neck neoplasms. Among the immune checkpoint inhibitors (ICI) are agents acting on cytotoxic T-cell lymphocyte-4 (CTLA-4) and programmed cell death-1/programmed cell death-ligand 1 (PD-1/PD-L1). Among Anti-CTLA-4 agents is ipilimumab, while among anti-PD-1 are pembrolizumab, nivolumab, and durvalumab, and among anti-PD-L1 are avelumab and atezolizumab.

Immunotherapy can result in immune-related adverse events (IrAEs) at the skin, gastroenterological, neurological, endocrinological, pulmonological, and cardiological levels. From a gastroenterological perspective, autoimmune diarrheal and colitis, i.e., immune-mediated diarrhoea and colitis (IMDC), can occur, the clinical manifestations of which include abdominal pain, nausea, diarrhoea, mucorrhea, rectorrhagia, peritoneal signs, up to and including conditions that can be life threatening [1,2]. Regarding frequency, gastrointestinal side effects occur most often in combination treatments between anti-CTLA-4 and anti-PD-1/PD-L1, followed by a single treatment with anti-CTLA-4 and a single therapy with anti-PD-1/PD-L1 [3].

Thus, side effects are mainly represented by inflammatory and autoimmune complications [4]. Indeed, ICI are monoclonal antibodies acting on the balance between immune system cells and tumour cells [4,5]. IrAEs can be highly variable, as mild, or life-threatening reactions are possible. The development of colitis is associated with an increased risk of ileus, toxic megacolon, and perforations [6].

From an endoscopic point of view, the colitis characteristics include the presence of erosions, ulcerations, erythema, reduction to loss of vascular pattern, mucosal friability, oedematous walls, and areas of exudation. An endoscopic negative examination (i.e., a normal colonic mucosa appearance) is also possible [7]. Faecal inflammatory markers, such as faecal calprotectin and lactoferrin, and serum markers, such as C-reactive protein, are helpful for risk stratification [8].

In patients refractory to steroid therapy, anti-tumour necrosis factor (TNF) α drugs such as infliximab can be administered by intravenous infusion, starting at 5 mg/Kg [6,9]. An alternative is treatment with vedolizumab, a monoclonal antibody acting on α_4_β_7_ integrin [9,10], administered as an intravenous infusion at a dose of 300 mg [6]. Faecal microbiota transplantation is another option for refractory patients [1,11]. Finally, surgery represents the last treatment option. An emergency colectomy is indicated in severe complications, such as perforation or toxic megacolon [11].

Ultimately, however, none of the major guidelines gives too much space to the introduction of Janus kinase (JAK)–signal transducer and activator of transcription (STAT) inhibitors as a therapeutic alternative by stigmatizing the still-needed evidence to prove it [12,13,14,15,16]. This review aims to provide the currently available evidence on the role of the JAK-STAT inhibitors in ICI colitis.

## 2. Immunotherapy, Generalities, and Toxicity: The Dimensions That Matter

The discovery of the basis of immunotherapy earned James Allison and Tasuku Honjo the Nobel Prize in Physiology or Medicine in 2018 [17].

Their research has elucidated the pivotal inhibitory role of PD-1 and CTLA-4 in immune function, revealing their capacity to effectively reinvigorate T cells and efficiently stimulate anti-cancer immunity [18].

CTLA-4 is a transmembrane receptor protein with inhibitory activity belonging to the immunoglobulin superfamily and localized on the surface of activated T lymphocytes [19]. Its physiological role is to regulate immune activity to prevent potential organ harm and immunity imbalances [19]. CTLA-4 expression on the lymphocyte’s surface appears in the early stages of its activation, approximately 48 h after the binding of costimulatory molecules B7-1 or B7-2 (also known as CD80 and CD86) to the CD28 receptor [19,20]. CTLA-4 competes with CD28 for binding with B7-1 and B7-2 [19,21]. Once CD28 is displaced, it binds to the costimulatory molecules, suppressing T-cell proliferation and survival signals [19]. This action occurs centrally within lymph nodes before T cells encounter tumour cells in the peripheral tissues and before any immune specificity develops [20,21].

Two monoclonal antibodies, ipilimumab and tremelimumab, can bind CTLA-4 and inhibit its normal function [22]. This inhibition prevents CTLA-4 from binding to B7-1 or B7-2 receptors, allowing these receptors to bind the co-stimulatory molecule CD28, leading to sustained activation of T lymphocytes. Consequently, this activation triggers an anti-tumour response [22].

On the other hand, PD-1 also belongs to the immunoglobulin superfamily, and it is found on the surface of activated T cells and pro-B cells [23,24]. It has a receptor function and binds two ligands (i.e., PDL-1 and PDL-2), which are part of the B7 costimulatory molecules expressed on antigen-presenting cells and epithelial cells [25]. The primary role of the PD-1-PDL-1 receptor–ligand interaction is to induce apoptosis in T cells activated against an antigen and enhance the efficiency of anti-apoptotic mechanisms in regulatory T cells [23,26]. This activity mainly occurs peripherally, within the tumour microenvironment, where activated T lymphocytes are already involved in immune responses. However, when they encounter tumour cells expressing PDL-1, their anti-tumour immune function is inhibited and disrupted [27]. Currently, in clinical practice, monoclonal antibodies targeting PD-1 include pembrolizumab, nivolumab, and cemiplimab, while those targeting PD-L1 include atezolizumab, avelumab, and durvalumab [28].

Interestingly, ICI can also be combined, as seen in the case of ipilimumab and nivolumab, for treating different tumour types such as oesophagus [29], lung [30], kidney [31], and melanoma [32]. Tremelimumab and durvalumab have recently gained approval for treating hepatocellular carcinoma [33].

Unfortunately, the management of immunotherapy using ICI is also burdened by a particular prevalence of adverse events related to this specific treatment. These adverse events are defined explicitly as irAEs. IrAEs have a higher incidence, especially when considering multiple ICI combined in the oncologic management of the patient [34].

Dermatologic irAEs are the most frequent but also among the earliest (i.e., they also arise more than two weeks after the start of treatment), with an incidence of about 44–68% in patients on anti-CTLA4 therapy and 37–42% in patients on anti-PD1 therapy and rates higher at 58% in patients on combination therapy [34]. Gastrointestinal tract toxicity, on the other hand, is pervasive in patients with melanoma, and lower gastrointestinal toxicity (i.e., ICI colitis) is prevalent with the use of anti-CTLA4 agents (i.e., 10–25% incidence) compared with anti-PD1 ICI (i.e., 1–5%) and, finally, with a considerable incidence when combination therapy is considered (i.e., about 20%) [34]. ICI can result in toxicity to other organs and systems. IrAEs encompass a large group of more than fifty different clinical entities that can affect almost any system, including the skin and gastrointestinal and the pulmonary, cardiovascular, hepatobiliary, and genitourinary systems [35].

There is often a delicate relationship between toxicity and oncologic response to immunotherapy. Khan et al., for example, reported how dermatologic toxicity (e.g., vitiligo, psoriasis) could predict oncologic response and overall survival in the case of bladder cancer [36].

In the literature, attempts have also been made to identify factors that could predict or, at the very least, quantify the risk of ICI colitis in patients undergoing ICI treatment. Wei et al. [37] conducted a meta-analysis reviewing clinical variables and identified several factors associated with a higher risk of ICI colitis, including combination therapy with ICI and chemotherapy and the use of anti-CTLA4 compared to anti-PD1 agents. Conversely, it appears that patients with non-small-cell lung cancer undergoing ICI treatment exhibit a lower risk of ICI colitis compared to patients with melanoma or other solid tumours. Additionally, it appears that in patients with renal cancer treated with ipilimumab, the chronic use of proton pump inhibitors may contribute to an increased risk of developing ICI colitis during treatment [38]. This reasoning highlights that the incidence of ICI colitis varies depending on the type of neoplasm under treatment. Specifically, the risk of ICI colitis is notably higher in melanoma (OR: 8.71) compared to patients with non-small-cell lung cancer (OR: 6.41) [37]. However, when considering all other tumours (such as small-cell lung cancer, prostate cancer, malignant mesothelioma, urothelial carcinoma, and head and neck cancer), it is essential to note that the subgroup analyses are less robust due to the limited availability of data, resulting in an odds ratio (OR) of 10.04 [37].

Among patient-related factors, comorbidities have also been taken into consideration. The primary comorbidity frequently examined is the presence of pre-existing inflammatory bowel disease (IBD). Some evidence suggests that, in patients with IBD, ICI colitis becomes a condition requiring more significant management attention, with a longer duration of the disease [39]. Interestingly, this does not appear to directly impact the oncological response [39]. Finally, additional efforts are required to identify genetic factors specifically associated with the onset of ICI colitis.

## 3. Clinical Evidence Concerning JAK-STAT Inhibitors and ICI Colitis

### 3.1. JAK Inhibitors: Generality and Classification

JAK inhibitors, over time, are classified according to their selectivity profile for different molecules of the JAK family.

**Figure 1:**
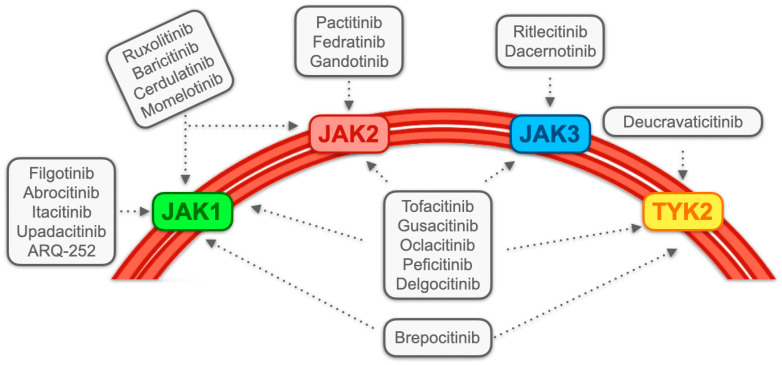
Main selectivity profiles of major Janus kinases (JAK) and JAK-related tyrosine kinase-2 (TYK2) inhibitors. Several JAK inhibitors and TYK2 inhibitors have been made over time. The broad affinity spectrum combining these molecules with JAK/TYK2 allows the blockade of an extensive range of immunologic phenomena aimed at blocking inflammation. The greater the selectivity profile, the greater the specific inhibition of the biological function to be controlled, as well as the spectrum of controlled pro-inflammatory cytokines. This underlies the wide variety of clinical indications that such molecules have received over time, encompassing different branches of medicine (e.g., gastroenterology, rheumatology, immunology, etc.).

In accordance with this definition, a diverse array of JAK inhibitors is identified, encompassing pan-JAK inhibitors such as tofacitinib, oclacitinib, and gusaticinib. The term “pan-JAK” implies the potential for these inhibitors to bind to all members of the JAK family. Additionally, there are JAK1,2-selective inhibitors like baricitinib and ruxolitinib, JAK1-selective inhibitors including filgotinib, upadacitinib, and itacitinib, and JAK2-selective inhibitors like gandotinib and fedratinib. Further classifications consist of JAK1/TYK2-selective inhibitors (e.g., brepocitinib), JAK3-selective inhibitors (e.g., ritlecitinib), and TYK2-selective inhibitors such as BMS-986165 and NDI-031301 [40,41] (see Figure 1).

JAK inhibitors have recognized a particular explosion in inflammatory diseases over time. They have been used in a broad spectrum of, for example, dermatologic diseases (such as alopecia areata, atopic dermatitis, erythema multiforme, vitiligo, and psoriasis), as well as in other inflammatory vascular disorders (such as polyarteritis nodosa), and rheumatologic and muscular diseases (such as rheumatoid arthritis, lupus erythematosus, hypereosinophilic syndrome, and dermatomyositis) [42].

A debated element on the use of JAK inhibitors has been that of safety. In particular, for some molecules (e.g., tofacitinib and upadacitinib), some red flags have been raised for cardiovascular events, venous thromboembolism, infections, and cancer; however, this issue must be related to the individual patient’s risk factors [43,44].

The use of JAK inhibitors, therefore, should be the focus of decision making that weighs the benefits and risks from the use of a moderate-to-severe immunosuppressant [45], taking into account the individual patient’s comorbidities and individual risk and the conventional and biologic therapy alternatives that are offered [46].

Certainly, JAK inhibitors are also gaining prominence in gastroenterology for managing IBD. While sharing commonalities with ICI colitis, the pathogenesis of IBD does exhibit differences, including a self-sustained inflammatory process characterized by a relapsing–remitting course independent of any previous patient treatment [47,48]. Despite this, these two pathologies share a wide range of molecular characteristics concerning the inflammatory pathways involved, as well as frequently exhibiting overlapping clinical and endoscopic presentations [49,50]. Consequently, IBD represents a valuable source from which potential therapeutic candidates for ICI colitis can be identified.

Tofacitinib [51,52], upadacitinib [53,54], and filgotinib [55] have now received indications of ulcerative colitis. Encouraging results appear to be provided in Crohn’s disease by upadacitinib [56,57] and filgotinib [58].

These results, produced from solid randomized clinical trials, demonstrate that in each case, JAK inhibitors can adequately control colonic inflammatory processes by leading to relevant rates of mucosal healing in settings of even moderate-to-severe intestinal inflammation, such as those of IBD. What is more, tofacitinib seems to be able to play a role even in cases of devastating inflammation, such as in acute severe ulcerative colitis, where its potential is increasingly emerging over the traditional use, in this setting, of parenteral steroids, cyclosporine, and infliximab [59,60,61,62].

Although these findings are not automatically applicable to ICI colitis precisely in light of the pathogenetic and etiologic differences between these entities, they nevertheless show a glimmer of the potential in ICI colitis of JAK-STAT pathway inhibition.

Some JAK inhibitors also showed encouraging results in managing other immune-mediated adverse events related to using ICI. Tofacitinib and baricitinib can attenuate the levels of monocyte chemoattractant protein-1 activated by using pembrolizumab, determining arthritic activation during treatment with ICI [63]. In detail, such activation at the level of synovial fluid determines the joint-level production of several cytokines, including TNFα [63]. A sound effect of baricitinib was also described in a complex case of pembrolizumab-induced arthritis in a patient with lung cancer and concomitant severe SARS-CoV-2 pneumonia [64].

### 3.2. Contemporary Management in Accordance with Established Guidelines and Exploration of Giverse Alternatives, with Limited Emphasis on JAK Inhibitors

Currently, ICI colitis is managed by a treatment approach proportional to the severity of the disease. A helpful model may be the European guidelines for ICI colitis [16]. 

According to this protocol, managing grade 1 ICI colitis (defined as an increase of up to 4 daily bowel movements from baseline) involves conservative measures such as a low-fibre diet, loperamide, and psyllium, while continuing ICI treatment. Should the escalation in bowel movements exceed 4–6 daily compared to baseline (grade 2), discontinuation of ICI therapy is recommended, and oral steroids at an immunosuppressive dose of 0.75–1 mg/kg daily should be incorporated into the treatment regimen. For more severe cases, specifically grade 3 (an increase of 7 or more bowel movements from baseline) or grade 4 (manifesting with severe symptoms like haematochezia, abdominal pain, mucus in stool, dehydration, or fever), consideration should be given to intravenous steroid therapy with methylprednisolone at a dose of 1 mg/kg/day. It is crucial to acknowledge the possibility of transitioning between grades.

In instances where grade 1 therapy proves ineffective after 15 days, a shift to grade 2 therapy is recommended. Similarly, if grade 2 therapy shows no response after 5–7 days, escalation to grade 3–4 treatment is advised.

In the event of recurrence post-steroid withdrawal or resistance to steroids (refractoriness), the introduction of biologic therapy becomes necessary, typically involving agents like infliximab or vedolizumab. Alternative options encompass faecal microbiota transplantation, ustekinumab, extracorporeal photopheresis, and, in extreme cases, colectomy.

Narrowing our attention to advanced therapies advocated by guidelines, namely biologic and small molecule treatments, it is projected that around 41% of patients exhibiting a certain responsiveness to infliximab may be suitable for this particular biologic [65]. Conversely, a non-negligible percentage, amounting to 11%, demonstrates explicit refractoriness to infliximab [65].

Vedolizumab, undoubtedly, represents a second option worth considering, particularly in cases of primary or secondary loss of response to infliximab. However, it is important to note that its association comes with the risk of a response that may not always be immediate, a criterion certainly desirable when dealing with ICI colitis beyond the second grade [66]. Nevertheless, it maintains a certain level of efficacy, especially in patients who were previously refractory to infliximab [66]. A recent meta-analysis has approximated the clinical remission rate (i.e., symptom disappearance) in patients with ICI colitis treated with vedolizumab at around 88% [67]. It is crucial to note that this estimate is based on a relatively limited study cohort, encompassing only 111 treated patients. Nevertheless, no significant differences were observed compared to infliximab.

Lastly, regarding ustekinumab, the available data is not yet firmly established, with a substantial portion still derived from case reports [68,69,70,71].

Even scarcer are the data assessing the recurrence rates of ICI colitis following appropriate treatment, as well as information pertaining to oncological outcomes, such as cancer progression or disease-free survival.

In addition, starting from grade 2 and onwards, it becomes imperative to conduct diagnostic tests aimed at excluding infections (such as *C. difficile* stool test, Cytomegalovirus, hepatitis viruses, tuberculosis test, and HIV). Additionally, an assessment of inflammation indices (C-reactive protein, faecal calprotectin, or faecal lactoferrin) is essential, culminating in the consideration of endoscopic evaluation [16].

These guidelines make reference to tofacitinib but do not allocate a central role in the treatment algorithm for severe, refractory, or steroid-dependent cases [15,16]. 

All these observations likely underscore the need to assess new treatments, incorporating oncological outcomes, particularly for patients who have not responded to the two available biologic therapies, for which we currently have more extensive data (i.e., infliximab and vedolizumab).

### 3.3. Navigating the Landscape: Unveiling Clinical Evidence from Pioneering Experiences to Ongoing Studies in JAK-STAT Pathway Inhibition for ICI Colitis

The major guidelines have not fully covered the use of JAK inhibitors in the treatment algorithm of ICI colitis [15]. Nevertheless, there have been reported instances of utilizing JAK inhibitors in this context. Generally, comprehensive studies with substantial sample sizes and meta-analyses for this indication are yet to be conducted.

Bishu et al. [72] reported the efficacy of tofacitinib in ICI colitis in a case series of four patients. Three of the patients had undergone treatment with a combination of ipilimumab/nivolumab for metastatic melanoma, while one patient received pembrolizumab and an indoleamine-pyrrole 2,3-dioxygenase inhibitor for adenocarcinoma of the lung (Table 1). Overall, the outcomes for all four patients were favourable, with recorded steroid-free remission. However, some necessitated a dose adjustment, revealing a dose-dependent response to tofacitinib. In nearly all the described cases, tofacitinib did not lead to changes in cancer prognosis. Patients who had achieved cancer remission prior to the initiation of tofacitinib maintained it weeks after commencing this JAK inhibitor. Furthermore, most patients exhibited an optimal response to tofacitinib, even if they had prior experience with infliximab. One patient, diagnosed with Crohn’s disease, had previously undergone treatments with ustekinumab and vedolizumab. In the remaining case, tofacitinib was employed with successful results as a first-line treatment for steroid-dependent ICI colitis.

Then, tofacitinib showed some efficacy in combination with faecal microbiota transplantation to treat an ICI enterocolitis in a 62-year-old male patient with metastatic melanoma treated with a variety of ipilimumab/nivolumab, adoptive cell therapy with tumour-infiltrating lymphocytes followed by ipilimumab/nivolumab and whole-brain radiotherapy [75]. Prior to commencing tofacitinib therapy and attaining clinical remission, the patient had experienced treatment failures with steroids, infliximab, antibiotics, loperamide, octreotide, opium, cholestyramine, and vedolizumab. Unfortunately, three weeks after being discharged from the hospital, the patient developed pneumonia with pulmonary embolism. This led to the discontinuation of tofacitinib, and the patient succumbed to cancer progression in the subsequent weeks.

In other experiences, however, faecal microbiota transplantation did not show the same efficacy. Sasson et al. [74] found rapid clinical resolution in a 61-year-old man with metastatic non-small-cell lung cancer who was refractory to other lines of therapy (intravenous corticosteroids, infliximab, and faecal microbiota transplantation). In detail, treating him with a tofacitinib dose of 10 mg twice daily was associated with a rapid clinical improvement with endoscopic and histologic remission in five weeks.

Sweep et al. [76] reported a similar case in a 67-year-old patient treated with ipilimumab/nivolumab and spinal palliative radiotherapy for metastatic melanoma who had developed ICI colitis and duodenitis. Once again, the patient did not achieve evident remission either with steroids or through the administration of biologics (infliximab and vedolizumab). This was observed even after an additional dose of 10 mg/Kg of infliximab, administered five days subsequent to a prior standard dose of 5 mg/Kg. Tofacitinib could control evacuation frequency and reduce indices of inflammation without altering the response to immunotherapy while preventing cancer progression.

A further similar case was reported by Esfahani et al. [73] in a gastric cancer patient treated with pembrolizumab, with ICI colitis refractory to treatment with corticosteroids, infliximab and vedolizumab. Treatment with tofacitinib induced clinical remission as early as five days, thus resulting in rapid normalization of bowel movements, maintaining the cancer response to immunotherapy in the subsequent months.

More recently, however, Sleiman et al. [77] reported the efficacy of tofacitinib in bringing about endoscopic healing of a case of ICI colitis by ipilimumab/nivolumab in a 68-year-old patient treated for metastatic adenocarcinoma of the transverse colon with no loss of response to immunotherapy during tofacitinib therapy.

Given the increasing demand for robust evidence generated through contemporary randomized controlled trials, the ongoing TRICK study (i.e., “An Open-label Study of Tofacitinib for the Treatment of Refractory Immune-related Colitis from Checkpoint Inhibitor Therapy”), identified by the clinicaltrials.gov code NCT04768504, is currently underway. This phase 2 open-label trial, presently in the recruitment phase and scheduled for completion in 2025, seeks to evaluate the efficacy of tofacitinib in treating ICI colitis. The outcomes of this trial are poised to significantly enhance our understanding of the subject.

Currently, there is a critical need for comprehensive evidence concerning other JAK inhibitors, particularly those that have already demonstrated efficacy in addressing intestinal inflammation, such as upadacitinib and filgotinib. This ongoing investigation serves to augment our knowledge, providing additional valuable insights into the potential applications of JAK inhibitors in managing ICI colitis.

### 3.4. The Hyperactivation of T Cells as a Potential Therapeutic Target for JAK Inhibitors: General Overview with a Focus on CD8^+^ Resident Memory T Cells

ICI colitis presents a pathogenesis that, unfortunately, remains not entirely elucidated, thereby posing the challenge of formulating definitive therapies that target all aspects of the disease’s pathogenesis. This challenge is particularly pronounced in the context of ensuring the continuity of immunotherapy, an essential component of the patient’s cancer treatment, and, consequently, prioritizing oncological outcomes in the therapeutic management of the patient.

It is evident that one pivotal event in the pathogenesis of ICI colitis is the hyperactivation of effector T cells. This is underscored by the presence of a clear infiltrate of CD4^+^ and CD8^+^ T cells in the colonic microenvironment of patients treated with anti-CTLA4 and anti-PD1, suggesting a significant role for these cells in the inflammatory process’s pathogenesis [78]. However, it appears that the stimulus for such cellular clones is ICI-related. Consequently, patients treated with anti-CTLA4 seem to predominantly express CD4^+^ T-cells, whereas conversely, those treated with anti-PD1 undergo a preferential selection of CD8^+^ T-cells [78]. It appears that, for these latter cell types (i.e., CD8^+^ T-cells), their origin can be traced back to the presence of resident memory T cells. This also explains the swiftness with which ICI colitis often manifests after the initial administration of immunotherapy [78].

**Figure 2:**
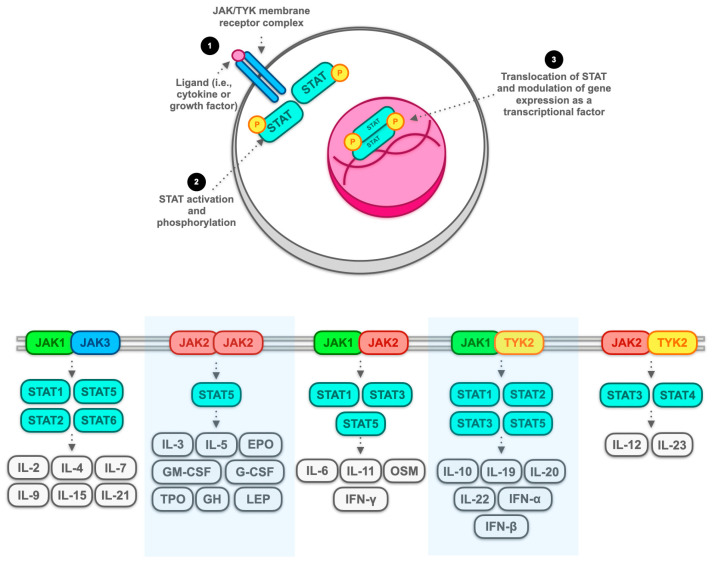
Cytokines and molecular mediators modulated by the Janus kinase (JAK) and tyrosine kinase (TYK2) system. The JAK/TYK2 system regulates the expression of numerous cytokines, growth factors, and hormones, resulting in a transcriptional crossroads of immune function and the inflammatory process. These kinases (i.e., JAK and TYK) interact with several transcription factors, namely those of the signal transducer and activator of transcription (STAT) family, which, through intranuclear translocation processes, regulate the gene expression of molecules that are part of the signalling pathway that combinations among all the mediators previously exposed determine. According to a simplified scheme (top of figure), activation of the membranous receptor complex consisting of the various combinations of JAK and TYK family members (bottom part of the figure) by binding of a ligand, which may be, for example, a cytokine or a transcription factor (1), which results in downstream activation, mainly by phosphorylation processes of STAT family members (2), which in turn are activated intranuclearly by acting as transcription factors and modulating gene expression of several genes coding for crucial molecules in the inflammatory process (3). Note: IL: interleukin; EPO: erythropoietin; GM-CSF: granulocyte–macrophage colony-stimulating factor; G-CSF: granulocyte-colony stimulating factor; TPO: thyroperoxidase; GH: growth hormone; LEP: leptin; OSM: oncostatin M; IFN: interferon.

As anticipated, the JAK-TYK2-STAT pathway has the potential to downstream regulate an endless array of key molecular mediators in the regulation of inflammatory processes [40,41,42] (see Figure 2). Moreover, it seems that this population of memory cells may represent a potential therapeutic target for tofacitinib, acting as a pan-JAK inhibitor.

Sasson et al. [74] showed a direct correlation between tofacitinib treatment and downregulation of JAK-STAT signalling and CD8^+^ T resident memory cell activation in lung non-small-cell carcinoma patients treated with carboplatin/pemetrexed and pembrolizumab. In detail, before the introduction of tofacitinib, widespread activation of CD4^+^ and CD8^+^ T cell clones with the maximum activation peak at CD8^+^ CD103^+^ T resident memory cells (about 61%) was detectable.

In-depth examination using flow cytometry on live CD45^+^ CD3^+^ T cells revealed a widespread activation of CD4^+^ and CD8^+^ T cells prior to the administration of tofacitinib, particularly within the CD8^+^ CD103^+^ T resident memory cell subset. Following a 6-week course of tofacitinib, a significant reduction in T cell activation was observed, encompassing the CD8^+^ CD103^+^ T resident memory cell subset.

**Figure 3:**
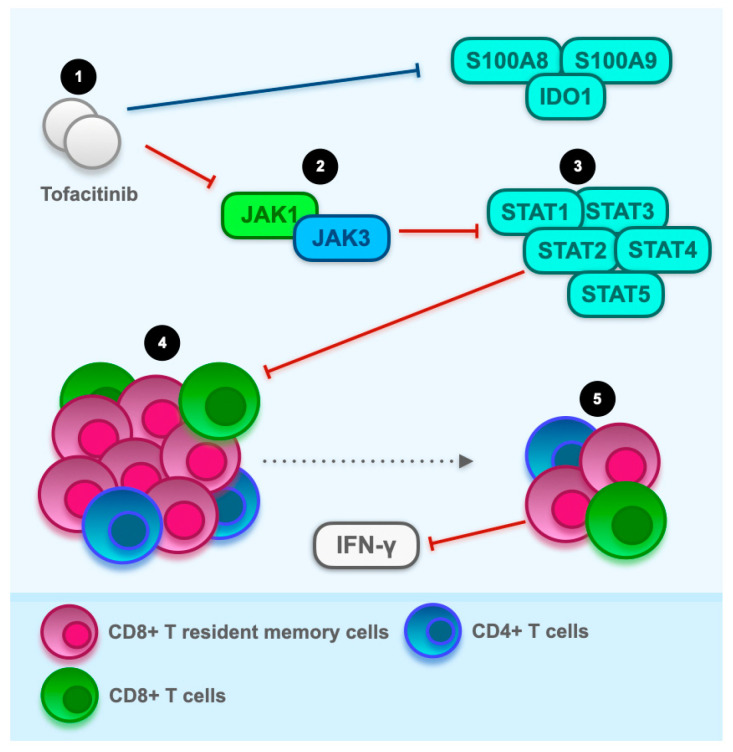
Hypothesis on the modulation of CD8^+^ CD103^+^ T resident memory cells by tofacitinib in immune checkpoint inhibitors (ICI) colitis. Drawing from a case involving a non-small cell lung cancer patient undergoing combination therapy (carboplatin/pemetrexed and pembrolizumab), this hypothesis suggests that tofacitinib, as a pan Janus kinase (JAK) inhibitor, may address the dysregulation of immunotherapy-induced CD8^+^ CD103^+^ T resident memory cell activation in the colonic microenvironment of ICI colitis patients. In the pre-therapy microenvironment with tofacitinib, CD8^+^ and CD4^+^ T cells exhibit extensive activation. Among CD8^+^ cells, the most active subset is CD8^+^ CD103^+^ T resident memory cells. Tofacitinib therapy leads to a substantial reduction in this activation, surpassing a fivefold decrease. At the genetic level, down-regulated molecules include JAK1, JAK3, signal transducer and activator of transcription (STAT) 1, STAT2, STAT3, and STAT4, potentially involved in interferon-γ (IFN-γ) signalling activation. This modulation of immunological processes may correspond to the clinical and endoscopic improvement observed in ICI colitis following tofacitinib therapy. In addition, the comprehensive transcriptional reaction to the administration of tofacitinib showcases a notable decrease in the expression of transcripts, specifically encompassing S100 calcium-binding protein (S100)A8, S100A9, and indoleamine 2,3-dioxygenase 1, also known as IDO1 (the blue line at the top of the figure).

Gene set enrichment analysis of bulk RNA sequencing data identified an enrichment of the interferon (IFN)-γ signalling pathway in ICI colitis. Data from the nano string RNAplex assay indicated a substantial down-regulation of JAK1, JAK3, STAT1, STAT2, STAT3, STAT4, and STAT5A upon tofacitinib treatment (Figure 3). The RNAplex assay conducted on colonic mucosal RNA before and after tofacitinib therapy demonstrated a down-regulation of crucial JAK-STAT signalling components downstream of IFN-γ signalling, aligning with the observed molecular alterations.

Overall, the transcriptional response to tofacitinib therapy manifested as a down-regulation of specific transcripts, including S100 calcium-binding protein A8 (S100A8), indoleamine 2,3-dioxygenase 1 (IDO1), and S100 calcium-binding protein A9 (S100A9).

Moreover, S100A8/A9 are transcripts associated with the S100 protein family, consisting of a group of calcium-binding proteins that are generically expressed in heterodimeric form and constructively expressed in neutrophils and monocytes [79]. In the pathogenesis of inflammatory processes, they are linked to the recruitment of leukocytes and the stimulatory secretion of pro-inflammatory cytokines [79]. Furthermore, this group of proteins has also been investigated as a potential inflammatory biomarker in IBD [80].

On the other hand, IDO1 is an enzyme utilizing tryptophan that has been implicated in signalling pathways promoting cancer-associated tissue inflammation [81]. This is because it stimulates immunological tolerance to self-antigens of tumour cells, allowing them to survive by evading immune response mechanisms. Conversely, IDO1 promotes internal tumour neovascularization, further fostering tumour growth and survival [81]. Notably, this has positioned IDO1 as a potential therapeutic target in immunotherapy [82].

In addition, it is known that in the pathogenesis of ICI colitis, a prominent role is played by CD8^+^ T resident memory cells that express high levels of C-X-C chemokine receptor type 6 and 3 (CXCR6/CXCR3), whose ligands (i.e., CXCL9 and CXCL10, respectively) are upregulated by IFN-γ and TNF [83]. This may partly explain why downregulation of the JAK-STAT pathway by tofacitinib may strongly reduce the activity of these cell subsets.

CXCR6 also represents an attractive therapeutic target in ICI colitis as it has a direct role in the genesis of tumour metastasis by determining increased ease with which tumour cells acquire the ability to migrate, invade and metastasize and conversely reduce this potential when there is a silencing of this molecule [84].

The role of regulatory T cells is clearly another cornerstone in the pathogenesis, as their expression of CTLA4, when binding to the co-stimulatory molecules on T cells (i.e., CD28 and CD80/86), leads to T cell activation [85]. Conversely, the interaction between PD-1 and PD-L1 results in the downregulation of transcription factors like the factor forkhead box protein 3 (Foxp3), causing a further reduction in the immunoregulatory activities of regulatory T cells [86]. This interplay, when activated by anti-CTLA4 and anti-PD1/PD-L1, contributes to the genesis of a microenvironment within the intestines that is highly conducive to the onset of ICI colitis [48]. On the other hand, members of the JAK-STAT family have connections with Foxp3. Notably, TYK2 can impact the regulation of regulatory T cells, as observed in CD4^+^ T cells lacking TYK2 expression, resulting in the absence of Foxp3 expression and an inability to differentiate into regulatory T cells [87]. Additionally, TYK2 has been found to promote the differentiation of CD4^+^ cells into T helper 1 lymphocytes [88].

In summary, the precise mechanisms through which JAK inhibitors, particularly tofacitinib, exert control over ICI colitis remain inadequately elucidated despite the existence of preliminary clinical evidence. Further exploration is imperative to comprehensively understand the intricacies of these therapeutic interactions.

## 4. Conclusions

ICI colitis unfortunately represents a common adverse event among patients undergoing cancer immunotherapy. Existing guidelines do not clearly advocate for the utilization of tofacitinib and other JAK inhibitors for managing this condition. Nevertheless, five case reports/series studies have demonstrated promising results in employing JAK inhibitors, specifically tofacitinib, to address ICI colitis in patients. The majority of these individuals were aged over 50, with metastatic cancer, and had experienced treatment failures across various conventional (e.g., antibiotics and steroids) and advanced (e.g., infliximab and vedolizumab) modalities. Notably, in certain instances, tofacitinib exhibited commendable performance as a first-line treatment for steroid-dependent cases.

While these studies are pioneering in nature, their significance in broader investigations necessitates validation to establish efficacy and safety rates. Given that tofacitinib and other JAK inhibitors exert moderate to severe immunosuppressive effects, careful consideration of their use is crucial in relation to the progression of the underlying malignancy. The outcomes of previously published case reports, however, present mixed findings, though, overall, tofacitinib has not demonstrated a significant impact on oncologic outcomes.

In conclusion, JAK inhibitors emerge as genuine and potential therapeutic candidates for ICI colitis, thereby warranting evaluation in dedicated randomized controlled trials. The specific JAK or STAT implicated in ICI colitis remains unclear. Clarification on this matter will inform the precise utilization of targeted JAK-STAT inhibitors.

## Figures and Tables

**Table 1:** Use of Janus kinase (JAK) inhibitors in immune checkpoint inhibitors (ICI) colitis: the main reports.

First Author, Year, Reference	N.	Study Type	JAK Inhibitor	Cancer Treated (N.)	ICI Employed (N.)	ICI Colitis Outcome	Cancer Outcome (N.)
Esfahani et al., 2020 [73]	1	CR	Tofacitinib	Gastric cancer	Pembrolizumab	ICI colitis steroid-free remission	Cancer response
Bishu et al., 2020 [72]	4	CS	Tofacitinib	Melanoma (3); lung adenocarcinoma (1)	Ipilimumab/nivolumab (3); pembrolizumab/indoleamine-pyrrole 2,3-dioxygenase inhibitor (1)	ICI colitis steroid-free remission	Cancer response (3); cancer progression (1).
Sasson et al., 2021 [74]	1	CR	Tofacitinib	Lung non-small-cell carcinoma	Carboplatin/pemetrexed and pembrolizumab	ICI colitis steroid-free remission	No mention concerning cancer response interference of tofacitinib
Holmstroem et al., 2022 [75]	1	CR	Tofacitinib plus FMT	Melanoma	Ipilimumab/nivolumab	ICI colitis remission	Cancer progression
Sweep et al., 2023 [76]	1	CR	Tofacitinib	Melanoma	Ipilimumab/nivolumab		Cancer response
Sleiman et al., 2023 [77]	1	CR	Tofacitinib	Colonic adenocarcinoma	Ipilimumab/nivolumab	ICI colitis steroid-free remission	Cancer response

N.: sample size; CR: case report; CS: case series; FMT: faecal microbiota transplantation.
